# Beyond bendy joints: number of variant connective tissue features predicts neurodivergent characteristics in hypermobile individuals with anxiety

**DOI:** 10.1038/s44184-026-00214-5

**Published:** 2026-06-10

**Authors:** Lisa Quadt, Jenny Csecs, Georgia Savage, Geoff Davies, Parashar Ramanuj, Alan J. Hakim, Nick Grey, Hugo D. Critchley, Jessica A. Eccles

**Affiliations:** 1 Department of Neuroscience, Brighton and Sussex Medical School, Trafford Centre, Brighton, UK; 2 Sussex Partnership NHS Foundation Trust, Arundel Road, Worthing, UK; 3 Berkshire Healthcare NHS Foundation Trust, Bracknell, UK; 4 Royal National Orthopaedic Hospital, Stanmore, UK; 5 Division of Medicine, School of Medicine, Penn State University, University Park, Hershey, PA USA; 6 Department of Neurology, National Hospital for Neurology and Neurosurgery, Queen Square, University College London Hospitals NHS Foundation Trust, London, UK

**Keywords:** Anxiety, Autism spectrum disorders, Chronic pain, Translational research

## Abstract

The goal of this study was to determine whether the number of connective tissue features in hypermobility is associated with the level of neurodivergent characteristics and establish whether autonomic reactivity may be an explanatory factor in the relationship between variant connective tissue and neurodivergent characteristics. 99 adult participants were assessed for joint hypermobility syndrome/hypermobile Ehlers–Danlos-Syndrome and filled out screening questionnaires for autism and ADHD. 99% of participants met criteria for generalised joint hypermobility, and 57% for hypermobile Ehlers–Danlos–Syndrome. 47% of participants scored above the screening threshold for autism, and 20% for ADHD. All measures were significantly correlated. Level of autonomic reactivity (as measured by the Body Perception Questionnaire) mediated the relationship between the number of connective tissue features and neurodivergence, even after controlling for anxiety level. This shows that autonomic reactivity has a potential mechanistic role in the established link between variant connective tissue and neurodivergence, opening novel pathways for research and clinical care.

## Introduction

Neurodivergence (e.g., autism, attention deficit hyperactivity disorder (ADHD)) frequently co-occurs with poor mental and physical health-, with potentially devastating consequences for the well-being and quality of life of neurodivergent individuals of all ages^1,2^. Neurodivergent individuals have a decreased life expectancy of up to 30 years^3^, and experience substantial barriers to effective healthcare^4^. Insight into factors that contribute to the poor health status of neurodivergent people is therefore much needed.

One potential factor is the overrepresentation of hereditary connective tissue disorders in this population^5–7^. Variant connective tissue, often expressed as joint hypermobility, may be present in approximately 20% of the general population^8^, and in up to 51% of the neurodivergent population^9^. When joint hypermobility is experienced in combination with medical symptoms, a diagnosis of hypermobility spectrum disorder (HSD) (previously known as joint hypermobility syndrome (JHS)) or Ehlers–Danlos Syndrome (EDS), of which hypermobile EDS (hEDS) is by far the most common, may be present^10^.

A population-based matched cohort study (nationwide registry) in Sweden (*n* = 1771) showed that individuals with EDS are 7.4 times more likely to be autistic than a comparison group, and 5.6 times more likely to have an ADHD diagnosis than those without EDS^5^. Moreover, autistic children have a greater frequency of presence of generalised joint hypermobility compared to a matched group of non-autistic children^11^, and the frequency of presence of generalised joint hypermobility in children with ADHD (*n* = 86) is 74% compared to 13% of a comparison group^12^.

Variant connective tissue often underlies poor physical and mental health, including chronic pain and fatigue^8,13,14^, gastrointestinal difficulties^15^, gynaecological and obstetric problems^16,17^ and psychological health difficulties, particularly anxiety^5,18,19^.

Variant connective tissue also affects the function of the autonomic nervous system, and has been shown to link neurodivergence and dysautonomia mechanistically^9^. Neural interactions that support autonomic regulation include homeostatic reflexes and allostatic control, which are both tightly coupled to viscerosensory feedback signals from internal bodily organs; i.e., interoception^20^. A hallmark of neurodivergence is a difference in sensory processing, and this includes interoceptive differences^21^. Joint hypermobility is also associated with differences in sensory processing, which include paraesthesia, hyperaesthesia, hyperacusis and hyperosmia^22–24^. Interoception, therefore, may help frame potential associations between autonomic dysregulation, neurodivergence and variant connective tissue, where autonomic reactivity may be conceptualised as heightened sensitivity to interoceptive signals. While associations are apparent in the aforementioned research and case studies^25^, the relationship between joint hypermobility, autonomic and interoceptive dysfunction, and neurodivergence requires further investigation and understanding^18^.

In the current study, we used screening data from a sample of potential participants for a clinical trial of an interoceptive therapy for people with joint hypermobility and anxiety^26^. None of the participants had formal diagnoses of neurodivergent conditions. We aimed, first, to estimate the frequency of autism and ADHD by establishing in this cohort who scored above threshold for screening criteria for these neurodivergent conditions and, second, to test whether autonomic reactivity was a potential mechanistic factor in the relationship between variant connective tissue and neurodivergence.

## Methods

### Study design and participants

This study used screening data gathered during the assessment phase of the Altering Dynamics of Autonomic Processing Therapy (ADAPT) trial (ISRCTN17018615), which was a randomised controlled trial comparing two non-drug therapies for anxiety in people with joint hypermobility^26^. We assessed 99 adults (age ≥18 years) interested in taking part in the ADAPT trial. Initial inclusion criteria were a score of ≥2 points on a five-part joint hypermobility questionnaire^27^ at screening, or having a hypermobility-related diagnosis: Joint Hypermobility Syndrome (JHS), Hypermobility Spectrum Disorder (HSD), Ehlers–Danlos Syndrome (EDS), and having anxiety (scored ≥16 on the Beck Anxiety Inventory at screening). Exclusion criteria for the ADAPT trial included major psychiatric disorders (except co-occurring depression, as it is commonly seen in anxiety) and a previously confirmed diagnosis of a neurodevelopmental condition (e.g., autism or ADHD). For this sub-study, we used data from the full screening interview. All inclusion and exclusion criteria for the ADAPT trial are available in the published protocol^26^.

### Procedure

Participants were initially screened for eligibility via phone by a research assistant. If they met the initial criteria, they were referred to a research Clinical Psychologist to obtain informed consent and conduct the full screening interview, alongside the research assistant. Due to COVID-19 restrictions at the time of screening, this assessment was conducted by completing questionnaires online via Qualtrics and meeting with researchers over video-conferencing software. See published protocol^26^ for a complete list of assessment measures.

Ethical approval was obtained from the London – Bloomsbury Research Ethics Committee on 4 January 2019 (reference ^18^:/LO/1920, IRAS project ID: 248326). Written informed consent to participate was obtained from all participants.

### Outcomes

Variant connective tissue was assessed using the 5-part questionnaire (5PQ) for joint hypermobility^27^, (scores of ≥2), the Brighton Criteria for JHS, and the 2017 hEDS criteria^28^. Full diagnostic criteria, including all items, are displayed in Supplementary Table 1. The hEDS diagnostic checklist has 3 criteria which need to be met: Criterion 1 – Generalised Joint Hypermobility (i.e. Beighton score ≥5 in pubertal men and women to age 50 or ≥4 if over age 50), Criterion 2 – two or more features (A, B or C) must be present (i.e. ≥5/12 of feature A, e.g. ‘unusually soft or velvety skin’; feature B: positive family history of hEDS; ≥1 of feature C: 3 items e.g. ‘chronic, widespread pain for ≥3 months) and Criterion 3 – all three prerequisites must be met (e.g. ‘absence of unusual skin fragility, which should prompt consideration of other types of EDS’). It was not possible to determine the last item of Criterion 2A (aortic root dilatation with Z-score >+2) as participants did not undergo echocardiography as part of the study protocol^29^. The hypermobility assessments were administered as clinical examinations by a trained clinician/researcher. The sum of hEDS Criterion 2A items that were met by each participant was used to quantify the number of connective tissue features^29^.

The Ritvo Autism Asperger Diagnostic Scale-Revised (RAADS-R^30^, endorsed by NICE Clinical guideline [CG142]^31^) was used in combination with a clinical interview, as part of a screening process for autism; this involved assessing autistic characteristics across 4 domains: Social Relatedness, Circumscribed Interests, Language and Sensory Motor. It consists of 80 self-report statements (e.g. ‘I often use words and phrases from movies and television in conversations’), with four options to choose from per statement (i.e. ‘true now and when I was young’, ‘true now only’, ‘true only when I was younger than 16’ and ‘never true’). Scores range from 0 to 240, and the threshold of likely autism is ≥65 (sensitivity = 97%, specificity = 100%)^30^. The study’s patient and public involvement group considered the RAADS-R to be a more acceptable screening tool for a sample that was likely to include mostly people who identified as female. Scores from the RAADS-R were subsequently used in conjunction with the clinical interview with Clinical Psychologists as part of the trial eligibility assessment; autistic characteristics which warranted further investigation (i.e. total RAADS-R score ≥65)^32,33^ were explored and, if appropriate, participants were recommended to be referred for full autism assessments.

The Wender Utah Rating Scale (WURS), designed for adults to rate their own childhood behaviour^34^, was used to assess ADHD characteristics. This was combined with a clinical interview with a Clinical Psychologist, as part of our screening process for ADHD. The WURS has 61 self-report items, which are rated in relation to presence and severity during childhood (e.g. ‘acting without thinking, impulsive’ is rated on a 5-point Likert scale from ‘0 = not at all or very slightly’ to ‘4 = very much’), 25 of the items are totalled to produce an overall score. Scoring 46/100 or more is suggestive of ADHD (sensitivity = 86%, specificity = 99%^34^,), and further explored in the clinical interview. Where appropriate, participants were recommended to be referred for full ADHD assessments.

The Body Perception Questionnaire (BPQ^35^) subscale ‘Autonomic Nervous System Reactivity’ was used as a self-report measure for autonomic reactivity. The scale contains 27 items of sensations related to autonomic nervous system reactivity, e.g., “My heart often beats irregularly”, that are rated in terms of frequency on a five-point scale ranging from ‘never’ to ‘always’. Scores range between 27 and 108.

### Statistical analyses

Participants were assessed as to whether they met criteria for generalised joint hypermobility, JHS, and hEDS (including the number of connective tissue features in Criterion 2A^28^). Total scores from the RAADS-R (including from the four domains and as an overall score) and from the relevant 25 items on the WURS were evaluated against the clinical cut-offs. The ‘Autonomic Reactivity’ subscale was also totalled as part of the BPQ.

We ran separate bivariate correlation analyses of differential relationships between the number of connective tissue features (hEDS Criterion 2A), RAADS total and RAADS sensory motor scores, WURS total scores, and autonomic reactivity. Missing data were handled by excluding cases pairwise.

To examine potential explanatory associations between neurodivergence, connective tissue features, and autonomic reactivity, we performed separate mediation analyses using the PROCESS macro v3.5 for SPSS by Hayes^36^. The 95% bootstrapped confidence interval for the indirect effect was based on 5000 samples and considered significant if the bootstrapped confidence intervals did not cross zero. We ran two separate mediation analyses with RAADS sensory motor scores and WURS scores as outcome variables. In both models, the predictor variable was the number of connective tissue features, and the mediator variable was autonomic reactivity scores. To test for possible confounding effects, mediation analyses were repeated with anxiety levels (GAD-7 total score), psychiatric medication use (yes/no), and demographic variables (age, gender identity, level of education, ethnicity) as covariates.

### Missing data

All participants self-reported that they had hypermobility, and data were available for the RAADS (*n* = 96, 97%), WURS (*n* = 98, 99%) and BPQ (*n* = 98, 99%) as part of screening before the full clinical assessment. The majority of the sample completed full hypermobility assessments as part of the clinical assessment; a small number of participants did not complete the full hypermobility assessments due to being ineligible at screening before further assessment (i.e. Beck Anxiety Inventory score <16, *n* = 6). Given the low number of missing data, we used listwise exclusion.

### Patient and public involvement

This specific sub-study of the ADAPT trial was motivated by our ongoing Patient and Public Involvement (PPI) work. We regularly correspond with patients and members of the public who express the wish for more research on the intersection of connective tissue disorders/joint hypermobility and neurodivergence. For the ADAPT trial, a Lived Experience Advisory Panel (LEAP) was formed via Sussex Partnership NHS Trust to engage patients and the public in the design of the study, design of materials for ethical approval, and strategies for recruitment and dissemination^26^. Meetings were held regularly with the LEAP group, and informed the choice of assessment questionnaires used in this sub-study. LEAP members and other PPI contacts will support the dissemination of findings among interested members of the public in an accessible manner. The authorship team includes individuals with lived experience of hypermobility and neurodivergence.

## Results

### Participants

Ninety-nine participants were assessed as part of the ADAPT trial. Most participants identified as female (*n* = 90, 91%), six identified as male (6%), one participant identified as non-binary, one participant identified as gender fluid, and one participant identified as transgender male. The mean age of participants was 38.6 years (SD = 12). For all participant demographic details, see Table 1. At screening, participants who disclosed a formal diagnosis of autism or ADHD were excluded, as per the original trial protocol, which was focused on anxiety alone.

**Table 1:** Demographic information

Demographic information	Frequency	Percent
Gender identity		
Male	6	6%
Female	89	91%
Non-binary/gender fluid/transgender male	3	3%
No answer	1	1%
Highest level of education		
GCSE/Equivalent	8	8%
Sixth form/college	19	19%
Undergraduate degree	33	33%
Graduate degree	17	17%
Doctorate degree	5	5%
Other	7	7%
Prefer not to say	1	1%
No answer	9	9%
Ethnicity		
White – British	80	81%
White – Irish	1	1%
Other White background	4	4%
Asian/Asian British/Indian	1	1%
Asian/Asian British/Pakistani	1	1%
Mixed – White and Black British	1	1%
Other ethnic background	2	2%
No answer	9	9%
Psychiatric medication use		
Yes	42	42%
No	48	49%
No answer	9	9%

### Outcome measures – screening thresholds for hypermobility, autism and ADHD

Table 2 summarises outcome scores and the number and percentage of participants scoring above the threshold for JHS, hEDS, autism, and ADHD. Almost half of the participants scored above the clinical cut-off for likely autism (*n* = 45, Table 2). Following further assessment as part of the clinical interview, Clinical Psychologists recommended that 34 participants be referred for full autism assessments (34% of the total sample). One-fifth of participants scored above the clinical cut-off for likely ADHD (*n* = 20). Following further assessment as part of the clinical interview, Clinical Psychologists recommended that 14 participants be referred for full ADHD assessments (14% of the total sample). Within these groups, seven participants were recommended for both autism and ADHD assessments (7% of the total sample).

**Table 2:** Outcome scores

Outcome measure	Available data	Mean (SD)	Met clinical cut-off (where applicable)
RAADS-R total/240 (*n* = 96)	96 (97%)	73.4 (51.5)	45 (47%)
RAADS-R Sensory Motor domain/60 (*n* = 96)	96 (97%)	22.7 (14.3)	58 (60%)
RAADS-R Social Relatedness domain/117 (*n* = 96)	96 (97%)	33.5 (24.5)	45 (47%)
RAADS-R Circumscribed Interests domain/42 (*n* = 96)	96 (97%)	12.7 (10.9)	35 (37%)
RAADS-R Language domain/21 (*n* = 96)	96 (97%)	5.2 (4.7)	52 (54%)
WURS total/100 (*n* = 98)	98 (99%)	30.4 (18.2)	20 (20%)
BPQ – Autonomic reactivity score/108 (*n* = 98)	98 (99%)	59.3 (19.2)	N/A

Almost all participants scored ≥2 on the 5-point questionnaire^27^ (*n* = 90, 99% of all participants who completed this measure) – mean score was 3.9/5 (SD = 0.8). 92 (99%) of those assessed met the historical Brighton Criteria for Joint Hypermobility Syndrome, and 53 (57%) met diagnostic criteria for hEDS. Under the new classification, 43% had HSD. The average number of connective tissue features across participants (measured using Criterion 2A of the hEDS criteria) was 4.6 (SD = 1.9).

### Correlation analyses

The number of connective tissue features (hEDS criterion 2A) positively correlated with sensory sensitivities, total autistic and ADHD characteristics, and autonomic hyperactivity. Table 3 summarises correlation results.

**Table 3:** Correlation results

	Number of connective tissue features in hEDS criterion 2A	Autistic characteristics: RAADS total	Sensory sensitivities: RAADS sensory motor total	ADHD characteristics: WURS total
**Autistic characteristics: RAADS total**	0.270**			
**Sensory sensitivities: RAADS sensory motor total**	0.332**	0.907***		
**ADHD characteristics: WURS total**	0.413***	0.436***	0.488***	
**BPQ autonomic reactivity total**	0.451***	0.454***	0.557***	0.489***

**p < 0.01.

***p < 0.001.

### Mediation analyses

**Fig. 1:**
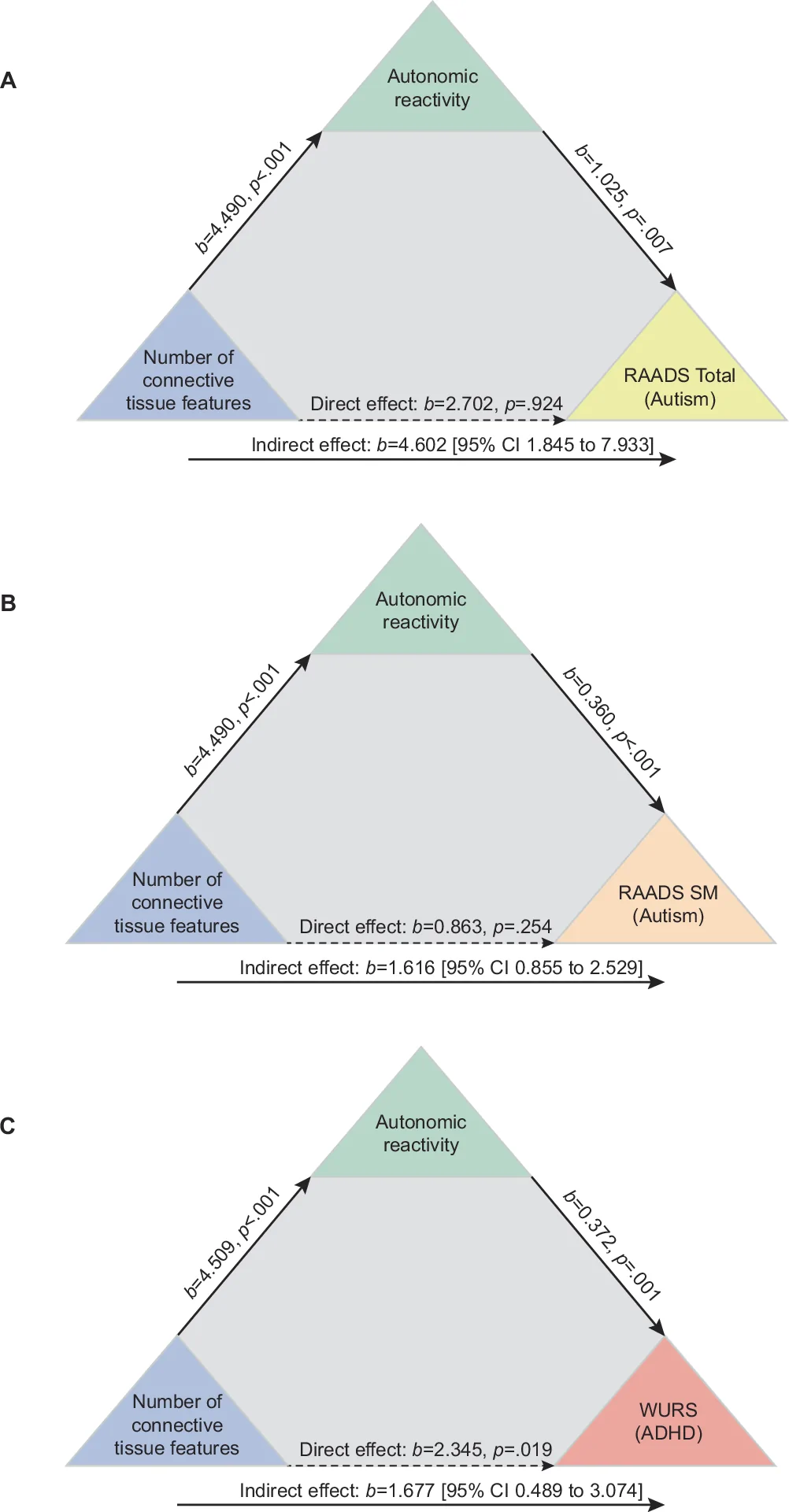
Mediation analyses. **A** Shows that autonomic reactivity mediates the relationship between the number of connective tissue features and scores on autistic characteristics (RAADS total). **B** Shows that autonomic reactivity mediates the relationship between the number of connective tissue features and RAADS sensory motor (RAADS-SM) sub-scores. **C** Shows that autonomic reactivity mediates the relationship between the number of connective tissue features and the Wender Utah Rating Scale scores as an index of ADHD traits.

The level of autonomic reactivity significantly mediated the relationship between the number of connective tissue features and sensory sensitivities, autism and ADHD characteristics (Fig. 1). This indicates that reactivity of the autonomic nervous system is a potential associative pathway in the relationship between hereditary connective tissue disorders and the presence of (undiagnosed) neurodivergence. These effects remained significant when correcting for anxiety level, psychiatric medication use, and demographic variables.

## Discussion

In this study, we showed that in hypermobile people who experience anxiety and have not previously been diagnosed with a neurodivergent condition, a high proportion are likely autistic and/or have ADHD. Furthermore, the number of variant connective tissue features predicted neurodivergent characteristics, autonomic hyperactivity and was particularly relevant for sensory sensitivities. Our findings support previous research showing the relationship between neurodivergence, anxiety, and variant connective tissue, adding nuance to this association, with autonomic reactivity as a possible explanatory factor. A recent study from our group further highlights the role of sensory processing^37^ in the relationship between neurodivergence, hypermobility and emotion regulation.

Although our mediation analyses were theoretically motivated by research indicating autonomic differences in hereditary connective tissue disorders^38–40^, the cross‑sectional design of this study precludes any strong inference about causal direction. The tested model represents one plausible pathway; however, alternative models—such as neurodivergent characteristics influencing autonomic regulation and sensory processing, thereby shaping the perception or reporting of connective tissue symptoms—are also highly plausible. Bidirectional relationships or shared developmental factors may underlie the observed associations. Future research should aim to disentangle the temporal and biological relationships between connective tissue variants, autonomic nervous system function, and interoceptive processing. Longitudinal and experimental studies are particularly needed to determine whether autonomic reactivity mediates the impact of connective tissue features on neurodivergence‑related traits, whether neurodivergent sensory and interoceptive profiles contribute to heightened awareness of hypermobility‑related symptoms, or whether shared developmental or neurobiological factors underlie all three domains. Investigating these dynamics with multimodal approaches—such as physiological monitoring, neuroimaging of interoceptive networks, and genetic or connective‑tissue phenotyping—may clarify how these systems interact and reveal potential targets for intervention.

Given that our participants were recruited specifically because they reported both marked anxiety and joint hypermobility, the sample reflects individuals with more complex or intense symptom profiles than would be found in the wider population. Although controlling for anxiety helped to account for some overlapping influences, other clinically relevant factors—such as mood symptoms, medication effects, chronic pain, sleep difficulties, or autonomic conditions—were not fully captured in our dataset and may have shaped participants’ responses. These considerations mean that the mediation pattern we observed should be interpreted cautiously, and future work with more diverse cohorts and broader clinical characterisation will be essential for clarifying these relationships.

Autism and ADHD affect approximately 2%^41^ and 5%^42^ of the adult general population. Also, it is traditionally – and incorrectly – assumed that autism and ADHD are significantly over-represented in males. Our data suggests that within the adult general population, there is a subgroup of people with joint hypermobility, predominantly female, in whom autism and ADHD are over-represented compared to the general population; almost half (47%) met screening threshold criteria for autism, and 20% for ADHD. At the same time, joint hypermobility as measured by the tools used in this study is found more often in females than males, perhaps accounting for the strong female representation in the sample.

No comparison group of people without anxiety was included because of the nature of the clinical trial, which purposefully advertised for people with anxiety. We can therefore draw no conclusions on whether the same patterns and relationships we found in our data would be similar in people with less or no anxiety. Additionally, since recruitment started during the first COVID-19 lockdown, participants completed the RAADS-R online and had constrained opportunities to ask questions and probe details about individual items with a practitioner. This might have impacted results, although the clinical assessments via video call likely compensated for this shortcoming.

Given our sample was mostly female, the screening tools used in this study may not have captured all likely neurodivergent participants. While the RAADS-R is usually considered better at picking up female autism traits than traditional screening measures such as the Autism Quotient^43^, a recent study suggests that the RAADS-R may have limited predictive validity for receiving an autism diagnosis^44^.

A further limitation concerns potential measurement confounding arising from the use of the RAADS‑R in a sample of individuals with anxiety. The RAADS‑R includes items relating to social avoidance, sensory sensitivities, and somatic experiences, which may overlap with anxiety symptoms—particularly in individuals with heightened autonomic reactivity. Given that all participants were recruited on the basis of experiencing clinically significant anxiety, elevations in RAADS‑R scores may partially reflect anxiety‑related processes rather than autistic traits alone. Although we statistically adjusted for anxiety in our mediation models, such adjustments cannot fully disentangle shared variance at the item level.

Our results support previous research, which indicates common neural pathways underlying joint hypermobility, anxiety, and sensory (including interoceptive) processing. Within the brain, the insular cortex is often termed a ‘hub’ for interoception^20,45,46^, receiving afferent bodily information and contributing to efferent autonomic control^47^. The region is also implicated in normal affective processing^48–51^, and emotional symptomatology in psychiatric disorders^52–54^, notably anxiety^55–57^. Furthermore, altered insular function may itself reflect the underlying noisy integration of imprecise interoceptive signals in neurodivergent people^58^, perhaps leading to dysautonomic symptoms. In a sample of hypermobile and non-hypermobile participants, sensitivity to interoceptive signals mediated the relationship between anxiety and hypermobility, where hypermobile participants expressed increased neural reactivity to emotional stimuli in the insular cortex^59^.

While the link between neurodivergence, variant connective tissue, and symptoms of dysautonomia is established^9^, the potentially explanatory role of autonomic reactivity observed in this study opens novel pathways for future research and clinical care. Training interoceptive abilities can reduce anxiety in autistic adults^60^, and interoception-based interventions are potentially promising for a wider range of mental health conditions^61^. New treatment possibilities may thus emerge to decrease autonomic reactivity by training interoceptive accuracy and thereby reducing noisy interoceptive signalling, which likely underlies heightened autonomic reactivity.

Our findings emphasise the need for transdiagnostic screening practices to detect co-occurring neurodivergence and variant connective tissue and to pay particular attention to the role of sensory hypo- and hyper-sensitivities. Integrated care for people with both conditions and improved education and awareness among healthcare professionals is needed to provide tailored care for a hitherto neglected population. It was beyond the scope of our study to assess whether participants screening positively for autism and ADHD traits received a diagnosis subsequently. A follow-up study could remedy this and help determine the accuracy of our screening tools. However, given that waiting lists in the UK are currently long, and diagnoses are not accessible for many individuals, novel screening instruments that are validated in diverse samples perhaps represent a more realistic strategic goal.

Our study highlights the need for research to determine the precise relationships between neurodivergence, variant connective tissue, and autonomic dysfunction. A focus on sensory processing and interoception may particularly offer insight into these relationships and reveal targets for intervention.

### Data sharing

The datasets generated and/or analysed during the current study are not publicly available due to data protection requirements, but are available from the corresponding author on reasonable request.

## Supplementary information

Supplementary Table 1

## Data Availability

The datasets generated and/or analysed during the current study are not publicly available due to data protection requirements, but are available from the corresponding author on reasonable request.

## References

[1] Ayres, M. et al. A systematic review of quality of life of adults on the autism spectrum. *Autism* **22**, 774–783 (2018). 10.1177/1362361317714988 28805071

[2] Asherson, P. ADHD across the lifespan. *Medicine* **48**, 737–741 (2020).

[3] Hirvikoski, T. et al. Premature mortality in autism spectrum disorder. *Br. J. Psychiatry* **208**, 232–238 (2016). 10.1192/bjp.bp.114.160192 26541693

[4] Doherty, M. et al. Barriers to healthcare and self-reported adverse outcomes for autistic adults: a cross-sectional study. *BMJ Open* **12**, e056904 (2022). 10.1136/bmjopen-2021-056904 PMC8883251 35193921

[5] Cederlöf, M. et al. Nationwide population-based cohort study of psychiatric disorders in individuals with Ehlers–Danlos syndrome or hypermobility syndrome and their siblings. *BMC Psychiatry*10.1186/s12888-016-0922-6 (2016). PMC4932739 27377649

[6] Dogan, ŞK., Taner, Y. & Evcik, D. Benign joint hypermobility syndrome in patients with attention deficit/hyperactivity disorders. *Arch. Rheumatol.* **26**, 187–192 (2011).

[7] Casanova, E. L., Baeza-Velasco, C., Buchanan, C. B. & Casanova, M. F. The Relationship between autism and Ehlers-Danlos Syndromes/hypermobility spectrum disorders. *J. Pers. Med.* 10.3390/jpm10040260 (2020). PMC7711487 33271870

[8] Mulvey, M. R. et al. Modest association of joint hypermobility with disabling and limiting musculoskeletal pain: results from a large-scale general population–based survey. *Arthritis Care Res.* **65**, 1325–1333 (2013). 10.1002/acr.21979 23401475

[9] Csecs, J. L. L. et al. Joint hypermobility links neurodivergence to dysautonomia and pain. *Front. Psychiatry*10.3389/fpsyt.2021.786916 (2022). PMC8847158 35185636

[10] Malfait, F. et al. The 2017 international classification of the Ehlers-Danlos syndromes. *Am. J. Med. Genet. C Semin. Med. Genet.* **175**, 8–26 (2017). 10.1002/ajmg.c.31552 28306229

[11] Shetreat-Klein, M., Shinnar, S. & Rapin, I. Abnormalities of joint mobility and gait in children with autism spectrum disorders. *Brain Dev.* **36**, 91–96 (2014). 10.1016/j.braindev.2012.02.005 22401670

[12] Shiari, R., Saeidifard, F. & Zahed, G. Evaluation of the prevalence of joint laxity in children with attention deficit/hyperactivity disorder. *Ann. Paediatr. Rheumatol.* **2**, 78 (2013).

[13] Hakim, A., De Wandele, I., O’Callaghan, C., Pocinki, A. & Rowe, P. Chronic fatigue in Ehlers–Danlos syndrome—Hypermobile type. *Am. J. Med. Genet. Part C: Semin. Med. Genet.* **175**, 175–180 (2017). 10.1002/ajmg.c.31542 28186393

[14] Voermans, N. C. & Knoop, H. Both pain and fatigue are important possible determinants of disability in patients with the Ehlers-Danlos syndrome hypermobility type. *Disabil. Rehabil.* **33**, 706–707 (2011). 10.3109/09638288.2010.531373 21077749

[15] Fikree, A., Chelimsky, G., Collins, H., Kovacic, K. & Aziz, Q. Gastrointestinal involvement in the Ehlers-Danlos syndromes. *Am. J. Med. Genet. Part C Semin. Med. Genet.* **175**, 181–187 (2017). 10.1002/ajmg.c.31546 28186368

[16] Hugon-Rodin, J., Lebègue, G., Becourt, S., Hamonet, C. & Gompel, A. Gynecologic symptoms and the influence on reproductive life in 386 women with hypermobility type Ehlers-Danlos syndrome: a cohort study. *Orphanet J. Rare Dis.* 10.1186/s13023-016-0511-2 (2016). PMC5020453 27619482

[17] Pezaro, S., Pearce, G. & Reinhold, E. Hypermobile Ehlers-Danlos syndrome during pregnancy, birth and beyond. *Br. J. Midwifery* **26**, 217–223 (2018).

[18] Bulbena, A. et al. Psychiatric and psychological aspects in the Ehlers-Danlos syndromes. *Am. J. Med. Genet. Part C Semin. Med. Genet.* **175**, 237–245 (2017). 10.1002/ajmg.c.31544 28186381

[19] Sharp, H. E. C., Critchley, H. D. & Eccles, J. A. Connecting brain and body: transdiagnostic relevance of connective tissue variants to neuropsychiatric symptom expression. *World J. Psychiatry* **11**, 805 (2021). 10.5498/wjp.v11.i10.805 PMC8546774 34733643

[20] Quadt, L., Critchley, H. & Nagai, Y. Cognition, emotion, and the central autonomic network. *Auton. Neurosci.* **238**, 102948 (2022). 10.1016/j.autneu.2022.102948 35149372

[21] Proff, I., Williams, G. L., Quadt, L. & Garfinkel, S. N. Sensory processing in autism across exteroceptive and interoceptive domains. *Psychol. Neurosci.* 10.1037/pne0000262 (2021).

[22] Hamonet, C. et al. Les multiples douleurs du syndrome d’Ehlers-Danlos. Description et proposition d’un protocole thérapeutique. *Douleurs Eval. Diagn. Trait.* **15**, 264–277 (2014).

[23] Colombi, M., Dordoni, C., Chiarelli, N. & Ritelli, M. Differential diagnosis and diagnostic flow chart of joint hypermobility syndrome/Ehlers-Danlos syndrome hypermobility type compared to other heritable connective tissue disorders. *Am. J. Med. Genet. Part C Semin. Med. Genet.* **169**, 6–22 (2015). 10.1002/ajmg.c.31429 25821090

[24] Baeza-Velasco, C., Grahame, R. & Bravo, J. F. A connective tissue disorder may underlie ESSENCE problems in childhood. *Res. Dev. Disabil.* **60**, 232–242 (2017). 10.1016/j.ridd.2016.10.011 27802895

[25] Sinibaldi, L., Ursini, G. & Castori, M. Psychopathological manifestations of joint hypermobility and joint hypermobility syndrome/ Ehlers-Danlos syndrome, hypermobility type: the link between connective tissue and psychological distress revised. *Am. J. Med. Genet. Part C Semin. Med. Genet.* **169**, 97–106 (2015). 10.1002/ajmg.c.31430 25821094

[26] Davies, G. et al. Altering Dynamics of Autonomic Processing Therapy (ADAPT) trial: a novel, targeted treatment for reducing anxiety in joint hypermobility. *Trials*10.1186/s13063-021-05555-4 (2021). PMC8453027 34548065

[27] Hakim, A. J. & Grahame, R. A simple questionnaire to detect hypermobility: an adjunct to the assessment of patients with diffuse musculoskeletal pain. *Int. J. Clin. Pr.* **57**, 163–166 (2003). 12723715

[28] Castori, M. et al. A framework for the classification of joint hypermobility and related conditions. *Am. J. Med. Genet. C Semin. Med. Genet.* **175**, 148–157 (2017). 10.1002/ajmg.c.31539 28145606

[29] Eccles, J. A. et al. Beyond bones: the relevance of variants of connective tissue (hypermobility) to fibromyalgia, ME/CFS and controversies surrounding diagnostic classification: an observational study. *Clin. Med.* **21**, 53–58 (2021). 10.7861/clinmed.2020-0743 PMC7850199 33479068

[30] Ritvo, R. A. et al. The Ritvo Autism Asperger Diagnostic Scale-Revised (RAADS-R): a scale to assist the diagnosis of autism spectrum disorder in adults: an international validation study. *J. Autism Dev. Disord.* **41**, 1076–1089 (2011). 10.1007/s10803-010-1133-5 PMC3134766 21086033

[31] NICE. *Autism spectrum disorder in adults: diagnosis and management (Clinical guideline [CG142]*), https://www.nice.org.uk/guidance/CG142/chapter/recommendations (2021). 32186834

[32] Nyrenius, J., Eberhard, J., Ghaziuddin, M., Gillberg, C. & Billstedt, E. Prevalence of autism spectrum disorders in adult outpatient psychiatry. *J. Autism Dev. Disord.* **52**, 3769–3779 (2022). 10.1007/s10803-021-05411-z 34993724

[33] Shaw, S. C. K., Doherty, M., Mccowan, S. & Eccles, J. A. Towards a neurodiversity-affirmative approach for an over-represented and under-recognised population: autistic adults in outpatient psychiatry. *J. Autism Dev. Disord.* **52**, 4200–4201 (2022). 10.1007/s10803-022-05670-4 35816244

[34] Ward, M. F., Wender, P. H. & Reimherr, F. W. The Wender Utah Rating Scale: an aid in the retrospective diagnosis of childhood attention deficit hyperactivity disorder. *Am. J. Psychiatry* **150**, 885–890 (1993). 10.1176/ajp.150.6.885 8494063

[35] Porges, S. *Body Perception Questionnaire* (Laboratory of Developmental Assessment, University of Maryland, 1993).

[36] Hayes, A. F. *Introduction to Mediation, Moderation, and Conditional Process Analysis: A Regression-Based Approach* 2nd edn (Guilford Publications, 2017).

[37] Eccles, J. A., Quadt, L., Garfinkel, S. N. & Critchley, H. D. A model linking emotional dysregulation in neurodivergent people to the proprioceptive impact of joint hypermobility. *Philos. Trans. R. Soc. Lond. B Biol. Sci.* **379**, 20230247 (2024). 10.1098/rstb.2023.0247 PMC11444222 39005028

[38] Csecs, J. L. L. et al. in *Frontiers in Psychiatry* Vol. 12 1–13 (Frontiers, 2022).

[39] De Wandele, I. et al. Dysautonomia and its underlying mechanisms in the hypermobility type of Ehlers-Danlos syndrome. *Semin. Arthritis Rheum.* **44**, 93–100 (2014). 10.1016/j.semarthrit.2013.12.006 24507822

[40] Gazit, Y., Nahir, A. M., Grahame, R. & Jacob, G. Dysautonomia in the joint hypermobility syndrome. *Am. J. Med.* **115**, 33–40 (2003). pii. 10.1016/s0002-9343(03)00235-3 12867232

[41] Dietz, P. M., Rose, C. E., McArthur, D. & Maenner, M. National and state estimates of adults with autism spectrum disorder. *J. Autism Dev. Disord.* **50**, 4258–4266 (2020). 10.1007/s10803-020-04494-4 PMC9128411 32390121

[42] Song, P. et al. The prevalence of adult attention-deficit hyperactivity disorder: a global systematic review and meta-analysis. *J. Glob. Health* **11**, 04009 (2021). 10.7189/jogh.11.04009 PMC7916320 33692893

[43] Baron-Cohen, S., Wheelwright, S., Skinner, R., Martin, J. & Clubley, E. The autism-spectrum quotient (AQ): evidence from asperger syndrome/high-functioning autism, malesand females, scientists and mathematicians. *J. Autism Dev. Disord.* **31**, 5–17 (2001). 10.1023/a:1005653411471 11439754

[44] Jones, S. L., Johnson, M., Alty, B. & Adamou, M. The effectiveness of RAADS-R as a screening tool for adult ASD populations. *Autism Res. Treat.* **2021**, 1–6 (2021). 10.1155/2021/9974791 PMC8452438 34552768

[45] Craig, A. D. Interoception: the sense of the physiological condition of the body. *Curr. Opin. Neurobiol.* **13**, 500–505 (2003). 10.1016/s0959-4388(03)00090-4 12965300

[46] Critchley, H. D. Neural mechanisms of autonomic, affective, and cognitive integration. *J. Comp. Neurol.* **493**, 154–166 (2005). 10.1002/cne.20749 16254997

[47] Quigley, K. S., Kanoski, S., Grill, W. M., Barrett, L. F. & Tsakiris, M. Functions of interoception: from energy regulation to experience of the self. *Trends Neurosci.* **44**, 29–38 (2021). 10.1016/j.tins.2020.09.008 PMC7780233 33378654

[48] Critchley, H. D., Eccles, J. & Garfinkel, S. N. in *Handbook of Clinical Neurology* Vol. 117 (eds Buijs, R. M. & Swaab, D. F.) Ch. **6**, 59–77 (Elsevier, 2013). 10.1016/B978-0-444-53491-0.00006-7 24095116

[49] Critchley, H. D., Wiens, S., Rotshtein, P., Ohman, A. & Dolan, R. J. Neural systems supporting interoceptive awareness. *Nat. Neurosci.* **7**, 189–195 (2004). 10.1038/nn1176 14730305

[50] Singer, T., Critchley, H. D. & Preuschoff, K. A common role of insula in feelings, empathy and uncertainty. *Trends Cogn. Sci.* **13**, 334–340 (2009). 10.1016/j.tics.2009.05.001 19643659

[51] Zaki, J., Davis, J. I. & Ochsner, K. N. Overlapping activity in anterior insula during interoception and emotional experience. *Neuroimage* **62**, 493–499 (2012). 10.1016/j.neuroimage.2012.05.012 PMC6558972 22587900

[52] Murphy, J., Brewer, R., Catmur, C. & Bird, G. Interoception and psychopathology: A developmental neuroscience perspective. *Dev. Cogn. Neurosci.* **23**, 45–56 (2017). 10.1016/j.dcn.2016.12.006 PMC6987654 28081519

[53] Nord, C. L., Lawson, R. P. & Dalgleish, T. Disrupted dorsal mid-insula activation during interoception across psychiatric disorders. *Am. J. Psychiatry* **178**, 761–770 (2021). 10.1176/appi.ajp.2020.20091340 PMC7613124 34154372

[54] Tran The, J., Magistretti, P. J. & Ansermet, F. Interoception disorder and insular cortex abnormalities in schizophrenia: a new perspective between psychoanalysis and neuroscience. *Front. Psychol.* 10.3389/fpsyg.2021.628355 (2021). PMC8281924 34276464

[55] Ernst, J. et al. The association of interoceptive awareness and alexithymia with neurotransmitter concentrations in insula and anterior cingulate. *Soc. Cogn. Affect Neurosci.* **9**, 857–863 (2014). 10.1093/scan/nst058 PMC4040102 23596189

[56] Gray, M. A., Harrison, N. A., Wiens, S. & Critchley, H. D. Modulation of emotional appraisal by false physiological feedback during fMRI. *PLoS ONE* **2**, e546 (2007). 10.1371/journal.pone.0000546 PMC1890305 17579718

[57] Paulus, M. P. & Stein, M. B. An insular view of anxiety. *Biol. Psychiatry* **60**, 383–387 (2006). 10.1016/j.biopsych.2006.03.042 16780813

[58] Bonaz, B. et al. Diseases, disorders, and comorbidities of interoception. *Trends Neurosci.* **44**, 39–51 (2021). 10.1016/j.tins.2020.09.009 33378656

[59] Mallorqui-Bague, N. et al. Neuroimaging and psychophysiological investigation of the link between anxiety, enhanced affective reactivity and interoception in people with joint hypermobility. *Front. Psychol.* **5**, 1162 (2014). 10.3389/fpsyg.2014.01162 PMC4196473 25352818

[60] Quadt, L. et al. Interoceptive training to target anxiety in autistic adults (ADIE): a single-center, superiority randomized controlled trial. *EClinicalMedicine* **39**, 101042 (2021). 10.1016/j.eclinm.2021.101042 PMC8350004 34401684

[61] Heim, N. et al. Psychological interventions for interoception in mental health disorders: a systematic review of randomized-controlled trials. *Psychiatry Clin. Neurosci.* **77**, 530–540 (2023). 10.1111/pcn.13576/full PMC7615164 37421414

