# Supplementary material for: Beyond bendy joints: number of variant connective tissue features predicts neurodivergent characteristics in hypermobile individuals with anxiety
Source: Npj Ment Health Res. 2026 Jun 10;5:51. doi: 10.1038/s44184-026-00214-5 (PMC13601541; doi:10.1038/s44184-026-00214-5)
Supplement: Supplementary file 1 [file 44184_2026_214_MOESM1_ESM.docx]

| **Diagnostic Criteria to evaluate symptomatic hypermobility** | |
| --- | --- |
| **2000 Revised Brighton Criteria for Joint Hypermobility Syndrome (JHS)** | |
| *JHS was diagnosed in the presence of two major criteria, or one major and two minor criteria, or four minor criteria. Two minor criteria sufficed where there was an unequivocally affected first-degree relative. Major 1 and Minor 1 are mutually exclusive as are Major 2 and Minor 2.* | |
| **Major criteria** | **Minor criteria** |
| 1. Current or historical Beighton score of ≥4 2. Arthralgia for longer than 3 months in 4 or more joints | 1. Beighton score of 1, 2 or 3 (0, 1, 2 or 3 if aged 50+) 2. Arthralgia (> 3 months) in one to three joints or back pain (> 3 months), spondylosis, spondylolysis/spondylolisthesis 3. Dislocation/subluxation in more than one joint, or in one joint on more than one occasion 4. Soft tissue rheumatism. > 3 lesions (e.g., epicondylitis, tenosynovitis, bursitis) 5. Marfanoid habitus (tall, slim, span/height ratio >1.03, upper: lower segment ratio less than 0.89, arachnodactyly [positive Steinberg/wrist signs] 6. Abnormal skin: striae, hyperextensibility, thin skin, papyraceous scarring 7. Eye signs: drooping eyelids or myopia or antimongoloid slant 8. Varicose veins or hernia or uterine/rectal prolapse |
| **2017 Hypermobile Ehlers Danlos Syndrome (hEDS) Criteria** | |
| *A clinical diagnosis of hEDS is made if all 3 criteria are met. Criteria 3 specifically excludes the diagnosis if features of other types of EDS/connective tissue disorders are suspected and/or adjusts requirements of Criterion 2 in the presence of acquired connective tissue disorder* | |
| **Criterion 1** | **Criterion 2 (Two or more of the following features (A, B, or C) must be present)** |
| Beighton score of   - ≥6 pre-pubertal children and adolescents - ≥5 pubertal men and woman to age 50 - ≥4 men and women over the age of 50   *If Beighton Score one point below age/ sex-specific cut off, Criterion 1 can be met if 2 or more questions are positive on the self-report scale of Hakim and Grahame (2003):*   1. Can you now (or could you ever) place your hands flat on the floor without bending your knees? 2. Can you now (or could you ever) bend your thumb to touch your forearm? 3. As a child, did you amuse your friends by contorting your body into strange shapes or could you do the splits? 4. As a child or teenager, did your shoulder or kneecap dislocate on more than one occasion? 5. Do you consider yourself “double jointed”? | A: (Five must be present)   - Unusually soft or velvety skin - Mild skin hyperextensibility - Unexplained striae distensae or rubae at the back, groins, thighs, breasts and/or abdomen in adolescents, men or pre-pubertal women without a history of significant gain or loss of body fat or weight - Bilateral piezo genic papules of the heel - Recurrent or multiple abdominal hernia(s) - Atrophic scarring involving at least two sites and without the formation of truly papyraceous and/or hemosideric scars as seen in classical EDS - Pelvic floor, rectal, and/or uterine prolapse in children, men or nulliparous women without a history of morbid obesity or other known predisposing medical condition - Dental crowding and high or narrow palate - Arachnodactyly, as defined in one or more of the following: (i) positive wrist sign (Walker sign) on both sides, (ii) positive thumb sign (Steinberg sign) on both sides - Arm span-to-height ratio ≥1.05 - Mitral valve prolapse (MVP) mild or greater based on strict echocardiographic criteria - Aortic root dilatation with Z-score >+2 |
|  | B:   - Positive family history: one or more first-degree relatives independently meeting the current criteria for hEDS |
|  | C: (≥ 1 feature must be present)   - Musculoskeletal pain in two or more limbs, recurring daily for at least 3 months - Chronic, widespread pain for ≥3 months - Recurrent joint dislocations or frank joint instability, in the absence of trauma |

***Supplementary Table 1: Diagnostic criteria to evaluate symptomatic joint hypermobility and hypermobile Ehlers-Danlos Syndrome***
